# Empirical use of temocillin in hospitalized patients: results from a retrospective audit

**DOI:** 10.1093/jacamr/dlad030

**Published:** 2023-04-19

**Authors:** Hala Kandil, Robert M Gray, Rakan El-Hamad, Madhuri Vidwans, Tejal Vaghela, Omar Naji, Sebastien Van De Velde

**Affiliations:** Microbiology Department, West Hertfordshire Teaching Hospitals NHS Trust, Vicarage Road, Watford, Hertfordshire WD18 0HB, UK; Department of Microbiology and Immunology, Faculty of Medicine, Alexandria University, Alexandria, Egypt; Microbiology Department, West Hertfordshire Teaching Hospitals NHS Trust, Vicarage Road, Watford, Hertfordshire WD18 0HB, UK; Department of Pathology and Microbiology, Faculty of Medicine, Jordan University of Science and Technology, Irbid, Jordan; Microbiology Department, West Hertfordshire Teaching Hospitals NHS Trust, Vicarage Road, Watford, Hertfordshire WD18 0HB, UK; Microbiology Department, West Hertfordshire Teaching Hospitals NHS Trust, Vicarage Road, Watford, Hertfordshire WD18 0HB, UK; Microbiology Department, West Hertfordshire Teaching Hospitals NHS Trust, Vicarage Road, Watford, Hertfordshire WD18 0HB, UK; Department of Medical Affairs, Eumedica s.a., Chemin de Nauwelette 1, 7170 Manage, Belgium

## Abstract

**Background:**

Following a global shortage of piperacillin/tazobactam in 2017, a formulary decision was taken at a large District General Hospital in the East of England to partly replace piperacillin/tazobactam with either temocillin as monotherapy or as part of a combination regimen. A retrospective audit was then conducted to assess the clinical effectiveness of temocillin therapy.

**Methods:**

Data from patients admitted to Watford General Hospital between May and August 2017 and treated with temocillin were reviewed retrospectively. Clinical characteristics of patients, data related to the episode of infection, clinical success, tolerance and mortality were analysed.

**Results:**

Temocillin was used in 126 patients with median age of 73 years. Infection episodes mostly originated from the abdomen (*n* = 46), the lung (*n* = 40) and the urinary tract (*n* = 21). Seventy-seven patients received temocillin as first-line therapy and 106 received it empirically, with temocillin prescribed in combination with another antibiotic in 82% of the empirically treated cases. Clinical success was observed in 88.9% of cases with no difference between patients treated empirically and others (89.6% versus 85%) or in efficacy among abdominal (91%), pulmonary (87.5%) and urinary (81%) infections. One case of *Clostridioides difficile* infection was reported in a patient treated with four different antibiotics. During the shortage period, the hospital’s standardized mortality ratio was significantly lower when compared with the same period of the preceding year (85 versus 96).

**Conclusions:**

Using temocillin as part of an empirical strategy is feasible and safe as long as appropriate antibiotic combination is recommended based upon the indication and the likely bacterial pathogen.

## Introduction

In October 2016, the explosion of a manufacturing site in China led to a major shortage of piperacillin/tazobactam across the globe. In response, the Department for Health and Public Health England (PHE) jointly issued guidance restricting the use of this antibiotic to specific indications only [neutropenic sepsis, severe sepsis of unknown source (SUS) and ventilator-associated pneumonia] and to place piperacillin/tazobactam use under the supervision of hospital microbiologists and Infectious Disease specialists.^1^ They also proposed alternative antibiotic regimens including the use of temocillin, a narrow-spectrum β-lactam active against the Enterobacterales but not against the anaerobes, Gram-positive bacteria or *Pseudomonas aeruginosa*. Temocillin was advised as monotherapy for pyelonephritis/urosepsis, in combination with metronidazole for complicated intra-abdominal infections (IAIs) and with amoxicillin and metronidazole for aspiration pneumonia. Combination therapy was advised to extend the limited bacterial coverage of temocillin and careful consideration of the likelihood of pseudomonal infection was advised in latter situations, including analysis of prior microbiology results, and gentamicin was added whenever *P. aeruginosa* was suspected.

In consequence, a formulary decision was taken in Watford General Hospital (520 beds) to replace piperacillin/tazobactam with temocillin (4 g daily or renally adjusted) alone for the empirical treatment of urinary tract infections (UTIs) and, where *Pseudomonas* was not expected, for hospital-acquired pneumonia (HAP) in combination with amoxicillin (1 g three times a day), for SUS in combination with amoxicillin (1 g three times a day) and metronidazole (500 mg IV three times a day) and, for IAI, in combination with metronidazole (500 mg IV three times a day). After the antibiotic formulary was changed, an audit was conducted to assess the efficacy and safety of this new local guidance.

## Methods

The audit was a service evaluation initiated at the request of the trust antimicrobial committee and conducted retrospectively for internal hospital guidance. According to the UK National Research Ethics Service, neither consent nor ethical approval was required.

During a 4 month period (May 2017 to August 2017), data were collected from each episode of infection treated with at least 3 days of temocillin. Patient demographics, renal function, microbiological data, prior and concomitant antibiotics, hospitalization time and clinical outcome were recorded. For analysis purposes, temocillin was categorized as first-line treatment if no previous antibiotic treatment was given to the patient and as second-line treatment if another antibiotic was given before starting temocillin treatment. Clinical success was defined as clinical and biochemical improvement with no need for escalation in antibiotics and no relapse requiring repeat treatment within 7 days of completing the antibiotic course, as assessed by a single reviewer, unless efficacy was unclear, in which case an assessment was also made by a second independent reviewer. Clinical failure was defined as the lack of clinical response as judged by the treating clinician and as evidenced by the NEWS score (with or without rising of inflammatory marker).

Antibiotic consumption (DDD/1000 bed days), healthcare-associated *Clostridioides difficile* infections (HA-CDI) and hospital standardized mortality ratio (HSMR) were calculated during the shortage period and compared with the same period in the preceding year. The HSMR was calculated by dividing the observed number of deaths at the hospital by the expected number of deaths, based on patient characteristics such as age, sex and diagnosis. A ratio above 100 means that more deaths are occurring at the hospital than would be expected, while a ratio below 100 means fewer deaths are occurring.

Statistical analyses were performed using JMP 14.0.0 (SAS Institute); continuous and nominal variables were compared using Student’s *t*-test and Fisher’s exact test, respectively.

## Results

### Demographic and baseline clinical data

One hundred and twenty-six episodes of infection from 126 patients were retrieved and included in the analysis. The mean age of patients was 70 years (SD 19 years) and ranged from 21 to 100 years. Forty-five patients had impaired renal function, i.e. estimated glomerular filtration rate (eGFR) < 60 mL/min/1.73 m^2^). Further patient demographics are presented in Table 1.

**Table 1.** Clinical characteristics of patients and type of infections

Characteristic	*n* (%)
Patients	126
Age (years), mean (±SD)	70 (±18.9)
Age (years), median	73
Renal function (mL/min/1.73 m^2^)	
eGFR > 60	81 (64.3)
30 < eGFR < 60	31 (24.6)
15 < eGFR < 30	12 (9.5)
eGFR < 15	2 (1.6)
Type of infections	
UTI	18 (14.3)
Urosepsis	3 (2.4)
HAP^a^	27 (21.4)
CAP	5 (4.0)
LRTI	8 (6.3)
IAI	46 (36.5)
Sepsis of intra-abdominal source	5 (4.0)
SUS	14 (11.1)

CAP, community-acquired pneumonia; LRTI, LRTI other than HAP and CAP.

a Two patients also had UTI.

### Characteristics of the infection episodes

Main infection episodes were from abdominal, pulmonary or urinary source (Table 1). Temocillin (alone or in combination) was used in the primary regimen in 77 episodes and as second-line therapy in 49 episodes. Purposes of its use as second-line therapy included sparing the use of piperacillin/tazobactam (*n* = 25), treatment escalation (*n* = 17) and de-escalation (*n* = 7). The antibiotic regimen was empirical in 106 episodes and pathogen-guided in 20, and when prescribed empirically, temocillin was combined with another antibiotic in 82% of cases (Table 2).

**Table 2.** Characteristics of temocillin prescriptions and clinical outcome

	*n* (%)^a^
Temocillin prescriptions (*N* = 126)	
First-line therapy (no previous treatment)	77 (61.1)
Empirically	65 (51.6)
Alone	11 (8.7)
In combination^b^	54 (42.9)
Pathogen-guided	12 (9.5)
Alone	4 (3.2)
In combination^b^	8 (6.3)
Second-line therapy (after another antibiotic)	49 (38.9)
Empirically	41 (32.5)
Alone	8 (6.3)
In combination^b^	33 (26.2)
Pathogen-guided	8 (6.3)
Alone	4 (3.2)
In combination^b^	4 (3.2)
Treatment duration by indication, mean ± SD	
UTI	4.6 ± 1.2
Urosepsis	9 ± 5.3
HAP^c^	7.7 ± 5.8
CAP	6.6 ± 2.2
LRTI	6.9 ± 2.2
IAI	5.6 ± 2.9
Sepsis of intra-abdominal source	5.6 ± 3.1
SUS	6.1 ± 2.8
Clinical success	112 (88.9)
Empirical (*n* = 106)	95 (89.6)
Pathogen-guided (*n* = 20)	17 (85)
By indication	
UTI	14 (77.8)
Urosepsis	3 (100)
HAP^c^	23 (85.2)
CAP	5 (100)
LRTI	7 (87.5)
IAI	42 (91.3)
Sepsis of intra-abdominal source	4 (80)
SUS	14 (100)

CAP, community-acquired pneumonia; LRTI, LRTI other than HAP and CAP.

a Unless otherwise stated.

b Antibiotic combination (up to three antibiotics) with temocillin included: metronidazole (*n* = 58), amoxicillin (*n* = 33), gentamicin (*n* = 19), co-amoxiclav (*n* = 5), teicoplanin (*n* = 3), clarithromycin (*n* = 3), flucloxacillin (*n* = 1), meropenem (*n* = 1).

c Two patients also had UTI.

Temocillin prescription was in line with local guidance (correct indication, correct combination if any and correct dosage) in 90 episodes (71%). Guidance from a microbiologist initiated the use in 26 episodes and the remaining 10 episodes were out of the scope of the local guidance without advice from the Microbiology Department.

Mean duration of treatment was of 6.2 ± 3.7 days, ranging from 3 to 34 days. Median duration is 5 days, ranging from 4–7 days and IQR were 5 ± 3 days. Treatment duration differed slightly per indication, without reaching statistical significance (Table 2).

### Microbiological data

Clinically significant positive microbial identification was obtained in 45 out of 126 episodes (36%) with the most common pathogens being *Escherichia coli* (*n* = 23), *Staphylococcus* spp. (6), other coliforms (*n* = 5), *Klebsiella pneumoniae* (*n* = 4), anaerobes (*n* = 3), *Candida* spp. (*n* = 3), other Gram-positive aerobes (*n* = 2) and *Pseudomonas* spp. (*n* = 1).

### Clinical outcome and mortality

Clinical success was observed in 112 episodes (88.9%), clinical failure in 12 episodes (9.5%) and 2 episodes (1.6%) were assessed as undetermined (refer to Table 2). Failures were observed in various indications: three HAP, four IAI, one sepsis of intra-abdominal source, one lower respiratory tract infection (LRTI) and three UTI. Among the 15 episodes caused by a bacterial isolate intrinsically resistant to temocillin, 2 failures were reported: one UTI caused by *P. aeruginosa* and one IAI caused by *Staphylococcus epidermidis*. We found no treatment failures in the 10 episodes that were out of the scope of the guidance. In patients treated empirically with temocillin, clinical success rate was 89.6% and on univariate analysis no significant alteration of the clinical outcome was related to any parameter (presence of renal insufficiency, type of infection, presence of sepsis, treatment duration, empirical use/pathogen-guided, use according to guidance).

There were four deaths despite escalation to meropenem, and one patient died after withdrawal of antibiotic treatment. During the period, the HSMR was 85, far below the expected mortality when compared with the same period of the preceding year (96).

### Tolerance

Overall the drug was well tolerated, with just one case of HA-CDI. This instance occurred 4 weeks after the end of temocillin treatment in a patient who first received piperacillin/tazobactam and cefotaxime before temocillin combined with metronidazole. At the hospital level, a reduction of 50% was observed in the HA-CDI rate during the shortage period plus 3 months thereafter, compared with the same period in the preceding year.

### Antibiotic consumption

During the 4 month-long shortage period, there was a 29% increase in the total antibiotic consumption (DDD/1000 bed days) compared with the same period in the preceding year. At the same time, there was a 44% and 4858% increase in the use of meropenem and temocillin respectively, whilst there was a 64% decrease in the use of piperacillin/tazobactam.

## Discussion

During the shortage the increase in antibiotic consumption that we observed was due to the use of antibiotic combinations. Although meropenem was only recommended in neutropenic sepsis and in penicillin-allergic patients, its consumption rose, probably due to a lack of experience with temocillin. The implementation of our guidance had no detrimental effect on the HSMR. However, guidance documents were not always followed, which could have been due to lack of confidence in the recommended regimens, miscommunication of the contents of our guidance or the short notice period between their publication and actual implementation.

When used empirically, clinical success was reached in 90% of the patients, which is comparable to other studies. Heard *et al.*^2^ reported a clinical efficacy of 82.0% (50/61) for patients treated empirically with temocillin (20 LRTI, 38 UTI, 3 other infections). In a recent prospective randomized controlled trial, temocillin was compared with cefotaxime for the treatment of febrile UTI, which showed that the regimens were of comparable effectiveness, with both similar early (97% versus 94%, respectively) and late (97% versus 92%, respectively) clinical responses.^3^ This latter study is in line with the current Belgian guidelines, which recommend the empirical use of temocillin for several types of UTI.^4^

Our dataset includes 46 IAI, an indication poorly described in literature for the use of temocillin despite a few studies on its tissue penetration.^5–7^ For this indication our study shows a clinical success rate of 91.3% when combined with metronidazole, which is comparable to the results of one study looking at temocillin use in critically unwell patients.^8^

Out data showed a 50% decrease in HA-CDI, which may have been due to the fact that penicillin combination antibiotics (e.g. co-amoxiclav and piperacillin/tazobactam) are associated with a significant increase in the risk of HA-CDI.^9^ Interestingly temocillin is known for its low ecological impact,^3,10–12^ which might have helped in this regard.

This audit has several limitations. The retrospective nature of the data may have introduced bias and no comparator group was included, though the HSMR and antibiotic consumption could be compared with the same period of the previous year. Furthermore, only a limited number of patients included in the dataset had a positive microbiological culture; this could have been due to starting treatment prior to sample collection or to the unavailability of accessible sample types from which to obtain an isolate (e.g. in the case of IAIs).

In conclusion, the shortage of piperacillin/tazobactam was an opportunity to implement new antibiotic regimens in our hospital. Though guidance was not always followed, using temocillin as part of an empirical strategy seems feasible provided that an appropriate antibiotic combination is recommended taking into account the indication and appropriate pathogen coverage. Further prospective trials are needed to confirm these preliminary data, particularly if IAI is to be accepted as an indication for this agent. In the meantime, this audit allows sharing of lessons from hospital practice that could be helpful, particularly during future periods of antibiotic shortages.
