# Correction to: Empirical use of temocillin in hospitalized patients: results from a retrospective audit

**DOI:** 10.1093/jacamr/dlad069

**Published:** 2023-05-31

This is a correction to: Hala Kandil, Robert M Gray, Rakan El-Hamad, Madhuri Vidwans, Tejal Vaghela, Omar Naji, Sebastien Van De Velde, Empirical use of temocillin in hospitalized patients: results from a retrospective audit, *JAC-Antimicrobial Resistance*, Volume 5, Issue 2, April 2023, dlad030, https://doi.org/10.1093/jacamr/dlad030

In the originally published version of this manuscript, the affiliations of authors Rakan El-Hamad and Tejal Vaghela were incorrect.

Rakan El-Hamad's affiliation was given as ‘Microbiology Department, West Hertfordshire Teaching Hospitals NHS Trust, Vicarage Road, Watford, Hertfordshire WD18 0HB, UK’ instead of ‘Department of Pathology and Microbiology, Faculty of Medicine, Jordan University of Science and Technology, Irbid, Jordan’.

Tejal Vaghela's affiliations were given as ‘Microbiology Department, West Hertfordshire Teaching Hospitals NHS Trust, Vicarage Road, Watford, Hertfordshire WD18 0HB, UK’ and ‘Department of Pathology and Microbiology, Faculty of Medicine, Jordan University of Science and Technology, Irbid, Jordan’, instead of just ‘Microbiology Department, West Hertfordshire Teaching Hospitals NHS Trust, Vicarage Road, Watford, Hertfordshire WD18 0HB, UK’.

These errors have been corrected.

